# In-air hearing in Hawaiian monk seals: implications for understanding the auditory biology of Monachinae seals

**DOI:** 10.1007/s00359-021-01498-y

**Published:** 2021-06-18

**Authors:** Brandi Ruscher, Jillian M. Sills, Beau P. Richter, Colleen Reichmuth

**Affiliations:** 1 Department of Ocean Sciences, University of California Santa Cruz, Santa Cruz, CA 95064 USA; 2 Institute of Marine Sciences, Long Marine Laboratory, University of California Santa Cruz, Santa Cruz, CA 95060 USA; 3 Department of Ecology and Evolutionary Biology, University of California Santa Cruz, Santa Cruz, CA 95060 USA

**Keywords:** Endangered, Phocid, Audiogram, Critical ratio, Auditory anatomy

## Abstract

The auditory biology of Monachinae seals is poorly understood. Limited audiometric data and certain anatomical features suggest that these seals may have reduced sensitivity to airborne sounds compared to related species. Here, we describe the in-air hearing abilities of a Hawaiian monk seal (*Neomonachus schauinslandi*) trained to participate in a psychophysical paradigm. We report absolute (unmasked) thresholds for narrowband signals measured in quiet conditions across the range of hearing and masked thresholds measured in the presence of octave-band noise at two frequencies. The behavioral audiogram indicates a functional hearing range from 0.1 to 33 kHz and poor sensitivity, with detection thresholds above 40 dB re 20 µPa. Critical ratio measurements are elevated compared to those of other seals. The apparently reduced terrestrial hearing ability of this individual—considered with available auditory data for a northern elephant seal (*Mirounga angustirostris*)—suggests that hearing in Monachinae seals differs from that of the highly sensitive Phocinae seals. Exploration of phylogenetic relationships and anatomical traits support this claim. This work advances understanding of the evolution of hearing in amphibious marine mammals and provides updated information that can be used for management and conservation of endangered Hawaiian monk seals.

**Supplementary Information:**

The online version contains supplementary material available at 10.1007/s00359-021-01498-y.

## Introduction

The Hawaiian monk seal, *Neomonachus schauinslandi*, is a phocid (true seal) species endemic to the Northwestern and main Hawaiian Islands. There is significant conservation concern for this endangered marine mammal, with approximately 1400 individuals remaining in the wild (Carretta et al. 2017; Pacific Islands Fisheries Science Center 2020). Monk seals are unique in comparison to seals living at higher latitudes as they experience relatively stable environmental conditions and resources year round. For this reason, monk seals do not breed synchronously during a brief, predictable period each year like temperate and polar seals that show much stronger seasonality in behavior. Instead, Hawaiian monk seals have an unusually prolonged reproductive period spanning at least nine months at the population level (Miller and Job 1992). While parturition tends to occur during spring and summer, females can give birth throughout much of the year (Kenyon and Rice 1959; Johnson and Johnson 1984; Johanos et al. 1994). This enables males to continuously compete for access to dispersed females that apparently come into estrous within a few weeks of weaning their pups (Johnson and Johnson 1984; Atkinson and Gilmartin 1992; Johanos et al 1994).

From a phylogenetic perspective, it is noteworthy that monk seals—including the extant Hawaiian monk seal and Mediterranean monk seal (*Monachus monachus*)—have been separated from their nearest living relatives for about 12 million years (Higdon et al. 2007; Berta et al. 2018; Rule et al. 2020). A more complete understanding of their biogeography is just emerging from recently discovered fossil data (Rule et al. 2020), which indicate that monk seals primarily evolved in the southern hemisphere. Monk seals belong to the Monachinae lineage of phocid Carnivores (Family Phocidae), which split from its sister lineage of Phocinae seals more than 15 million years ago (Higdon et al. 2007; Berta et al. 2018; Rule et al. 2020). Monachinae seals are sometimes referred to as the clade of ‘southern’ seals and also include the elephant seals and Antarctic seals. These species have some anatomical differences from Phocinae seals, including with respect to their auditory anatomy. While all true seals have hypertrophied ossicles, cavernous tissue in the middle ear, and a muscular external ear opening that lacks a pinna (see Nummela 2008), Monachinae species have a much smaller external ear opening (King 1983) and a long and unsupported ear canal relative to Phocinae species (King 1969). Monachinae seals also have a relatively small fenestra vestibuli (oval window) compared to the size of the tympanum (King 1983). These features could indicate poor sensitivity to terrestrial sounds. Furthermore, monk seals exhibit certain ‘basal’ auditory traits, specifically with respect to tissues in the middle ear and the morphology of bony structures surrounding the inner ear (Repenning 1972; Repenning and Ray 1977; Wyss 1988). The retention of these traits suggests that the hearing abilities of monk seals may differ from those of related species.

Few audiometric studies have attempted to describe hearing in Monachinae seals. Rather, most auditory data are available for Phocinae species. These hearing profiles indicate a broad range of underwater hearing (< 0.1 to > 70 kHz) with best sensitivity near 50 dB re 1 µPa. The frequency range of hearing is narrower in air (< 0.1 to > 30 kHz) with best sensitivity as low as − 13 dB re 20 µPa (Reichmuth et al. 2013; Southall et al. 2019a), rivaling the best terrestrial carnivores (Fay 1988). There is a good understanding of amphibious auditory abilities in Phocinae species including harbor seals (*Phoca vitulina*), spotted seals (*Phoca largha*)*,* and ringed seals (*Pusa hispida*). Conversely, audiometric data are available for only two Monachinae species and both show relatively poorer hearing abilities. One northern elephant seal (*Mirounga angustirostris*) had an underwater hearing profile that was generally similar to that of the Phocinae seals but with somewhat elevated thresholds (Kastak and Schusterman 1999). Aerial measurements obtained with the same individual suggested reduced sensitivity to airborne sounds (Reichmuth et al. 2013). Hearing in Hawaiian monk seals has also been studied. An initial report by Thomas et al. (1990) suggested that monk seals perceive underwater sounds across a more restricted frequency range (~ 10–30 kHz) than other seals. In contrast, Sills et al. (2021) describe a much broader range of hearing in water (< 0.1 to > 60 kHz), although with sensitivity poorer than 73 dB re 1 µPa. No in-air audiometric data are available for Hawaiian monk seals.

Descriptions of vocal behavior provide some additional clues about the auditory biology of this species. Hawaiian monk seals emit low-frequency (< 1 kHz) vocalizations while on shore, which include mother–pup contact calls, threats, and other social vocalizations (e.g., Kenyon and Rice 1959; Miller and Job 1992; Job et al. 1995). While this species was previously thought to be silent under water, recent work has identified a moderate repertoire of low-frequency (< 1 kHz) vocalizations with apparent reproductive function that are produced by mature males (Sills et al. 2021). The generation of uniformly low-frequency sounds might suggest acute low-frequency hearing abilities in this species. This is apparently not true for Hawaiian monk seals in water, however, and it is unknown to what extent they can hear biologically relevant sounds in air.

In this study, we evaluated the in-air hearing sensitivity of a highly trained Hawaiian monk seal whose underwater hearing had previously been described (Sills et al. 2021). Using similar methods, we conducted in-air audiometric testing in a specialized acoustic chamber to allow for direct comparison to related species. We measured (1) absolute (unmasked) auditory thresholds across the range of hearing from 0.1 to 33.2 kHz, and (2) masked thresholds in the presence of spectrally flat, octave-band noise to reveal critical ratios at 0.8 and 3.2 kHz. Given the unique life-history characteristics, phylogenetic status, and auditory anatomy of Hawaiian monk seals, these measurements improve our understanding of sound reception in this species, provide updated guidance for management of noise-related disturbance, and, more generally, increase knowledge of auditory biology in Monachinae seals.

## Materials and methods

### Subject

**Fig. 1:**
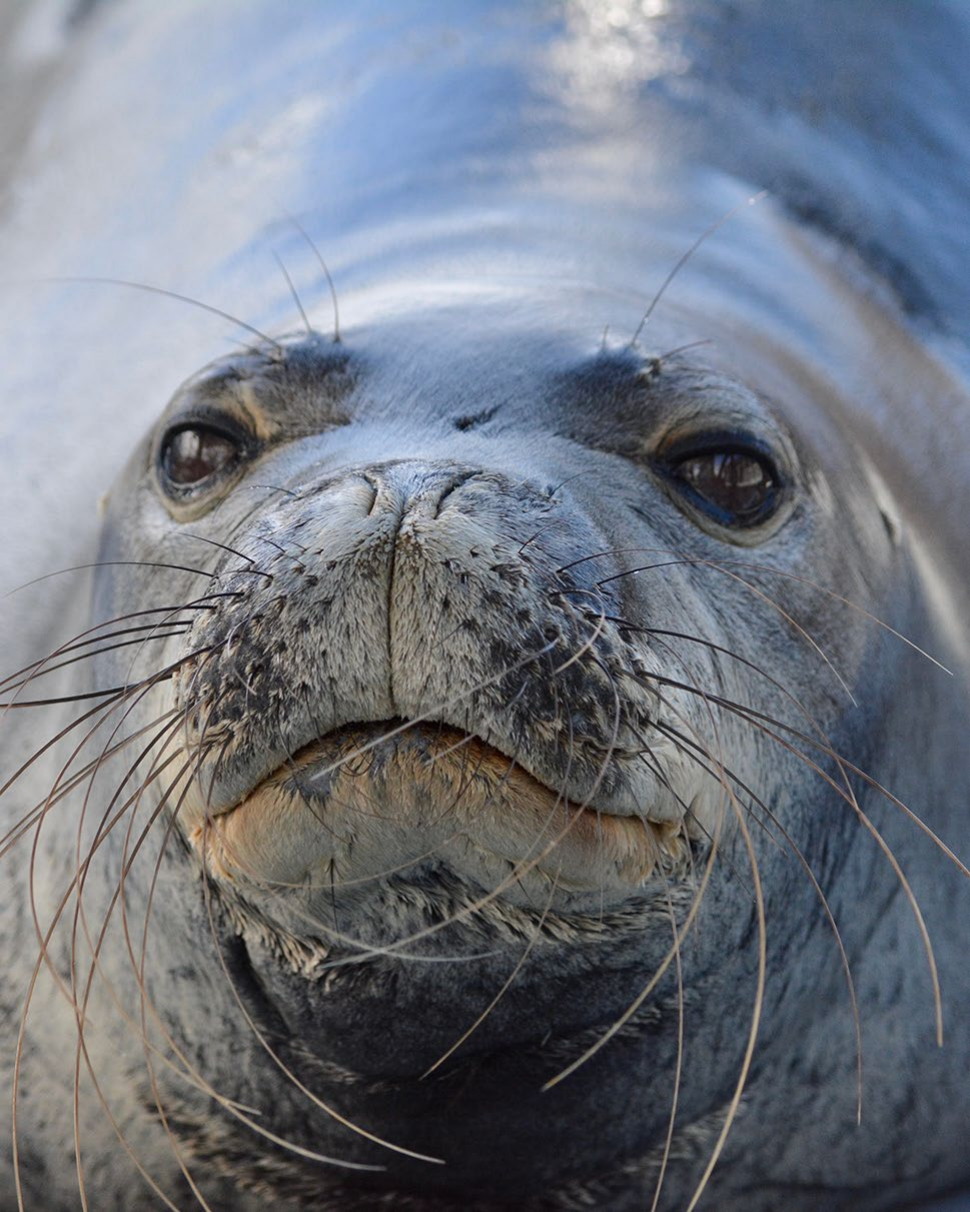
Hawaiian monk seal KE18. Photo collection authorized under NMFS permit 19590 Photo credit: C. Reichmuth

The subject was an adult male Hawaiian monk seal identified as KE18 (NOA0006781; also known as *Kaimalino* or *Kekoa*) (Fig. 1), who lived in the waters surrounding the Northwestern Hawaiian Islands for his first ten years of life. After exhibiting aberrant aggressive behavior towards conspecifics, he was removed from the wild by the US National Marine Fisheries Service and relocated to Long Marine Laboratory at the University of California Santa Cruz to participate in behavioral research to support conservation and management of the species. KE18 was in good health with no known history of ear injury or exposure to ototoxic medication. At the start of the study, he was 17 years of age and weighed approximately 200 kg. With respect to his external ears, curvilinear interaural distance was 26 cm (measured as the dorsal curvilinear length between meatal openings), while direct interaural distance (straight length) was 21 cm. Prior to this study, KE18 completed a similar behavioral assessment of underwater hearing (Sills et al. 2021).

KE18 was trained using operant conditioning methods and fish reinforcement for voluntary participation in husbandry and research sessions. He received one-third to one-half of his daily diet of freshly thawed fish and squid during audiometric sessions; his diet was not constrained for experimental purposes. The monk seal typically participated in one audiometric session per day, 5 days per week. Data collection occurred over a 12-month period beginning in April 2019.

### Testing environment

Audiometric testing was conducted in a sound-attenuating, hemi-anechoic acoustic chamber (Eckel Industries, Cambridge, MA, USA) that was located 30 m from the seal’s living enclosure. This custom chamber had a 3.3 × 2.3 × 2.2 m testing room with double paneled stainless-steel walls and ceiling lined with fiberglass-filled aluminum wedges. The floor of the chamber was covered with thick (> 2 cm) foam mats. Sessions were controlled by a technician from an adjacent, sound-isolated room and monitored in real time with a video surveillance system.

Ambient noise in the acoustic chamber was measured prior to each session in the absence of the animal. One-minute, unweighted measurements were obtained with a battery-powered 2250 or 2270 sound analyzer (Brüel and Kjær A/S, Nærum, Denmark) with a calibrated Brüel and Kjær 4189 free-field microphone (flat frequency response 0.006–20 kHz), which was placed at the location corresponding to the center of the monk seal’s head during testing. We calculated percentile statistics of 1/3-octave band noise levels from equivalent continuous sound pressure levels (*L*_eq_) for frequencies from 0.04 to 20 kHz. Power spectral density levels for the entire study period were then calculated from the median of daily 1/3-octave band 50th percentile measurements (L50) that included each test frequency. Equipment limitations prevented absolute noise measurements above 20 kHz.

### Audiometric procedure

We used cooperative behavioral methods to measure hearing sensitivity with a ‘go/no-go’ psychoacoustic procedure (Stebbins 1970) (Online Resource 1). After voluntarily leaving his home pool to enter the acoustic chamber with a trainer, KE18 placed his head on a polyvinyl chloride (PVC) listening station that ensured consistent positioning of his ears 0.3 m above the foam mat within a calibrated sound field. The trainer remained to the right of the seal, at least 1 m behind the station. A PVC response target was located 23 cm to the left of the station, and a light in front of the station indicated the 5-s window during which a signal could be presented. Each trial began when the monk seal was settled in the station and ended either when he touched the response target to indicate the presence of a signal or withheld this response for the full 5-s trial interval when he did not perceive a signal. Correct responses—remaining still at the station when no signal was present or touching the response target on signal-present trials—were marked with a conditioned acoustic reinforcer (buzzer) followed by primary reinforcement (fish) delivered by the trainer. Incorrect responses—remaining still on the station during a signal-present trial (miss) or touching the response target on a signal-absent trial (false alarm, FA)—were not reinforced and KE18 continued on to the next trial after a brief pause. The trainer received instructions via headphones and was unaware of individual trial conditions; even so, to prevent inadvertent cuing, the trainer was positioned out of the seal’s view on each trial.

An adaptive staircase method was used to estimate hearing threshold (Cornsweet 1962). For each session, frequency was held constant and signal amplitude was manipulated. Sessions began with signals presented at an easily detectable level (~ 20 dB above expected threshold), after which signal amplitude was decreased by 4 dB after each correct detection (hit) until the first miss. Signal amplitude was then increased by 4 dB following each miss and decreased by 2 dB following each correct detection. After five hit-to-miss transitions within a span of 6 dB, the signal was returned to an easily detectable level for multiple trials to maintain stimulus control at the end of the session. Sessions included 40–60 trials in a predetermined, pseudorandom order with signals presented on 50–70% of trials. This ratio was manipulated between sessions to maintain a consistent response bias over the study interval. False alarm rate was calculated as the number of FAs out of the total number of signal-absent trials during the ‘test phase’ of the session—that is, between the first and fifth consistent hit-to-miss transitions.

Testing was completed at each frequency when performance was stable, and the average miss level fell within 3 dB across three sessions. A psychometric function was fit to the proportion of correct detections at each signal level presented, and an inverse prediction was applied to determine threshold as the sound pressure level (SPL, dB re 20 μPa) corresponding to 50% correct detection (see Finney 1971). Threshold criteria required 95% confidence intervals to be less than 4 dB and corresponding FA rate to be above 0.0 and below 0.3. We tested frequencies to completion in random order, and repeated testing at two frequencies to ensure no learning effect over the course of the experiment.

### Absolute hearing thresholds

We measured auditory sensitivity at 11 frequencies across the range of hearing: in octave steps from 0.1 to 25.6 kHz and at 33.2 kHz, the highest frequency to which KE18 exhibited reliable responses. Additionally, we measured hearing at 18.1 kHz to complement his underwater audiogram (Sills et al. 2021). This frequency was of particular interest due to increased sensitivity noted under water in this region by Thomas et al. (1990).

Acoustic stimuli were 500 ms frequency modulated upsweeps with 10% bandwidth (± 5% from center frequency) and 5% rise/fall times, generated using Hearing Test Program (HTP) software (Finneran 2003). The stimuli were sent through a USB-6259 BNC M-series data acquisition module (update rate 500 kHz; NI, Austin, TX, USA), a 3364 anti-aliasing bandpass filter (Krohn-Hite, Brockton, MA, USA), and a PA5 digital attenuator (Tucker-Davis Technologies, Alachua, FL, USA) to the designated speaker in the acoustic chamber. Signals were projected through a 2245H speaker (JBL Incorporated, Northridge, CA, USA) for 0.1 kHz signals, a JBL 2123H speaker for 0.2–6.4 kHz signals, or a FT96H speaker (Fostex Company, Tokyo, Japan) for 12.8–33.2 kHz signals. These transducers were positioned 0.8–1.4 m in front of and on axis with the station. We determined speaker locations through spatial mapping of the sound field, which we conducted at each frequency to ensure that variability in received signals did not exceed ± 3 dB across 14 positions. The mapping grid included each ear and six points surrounding each ear with 2 cm spacing (forward/backward, left/right, and up/down from the ear position). This grid encompassed all possible locations of the external ears during testing.

Signals were calibrated daily at the location of the external ear that had the higher received level during spatial mapping. Signals were measured at a range of amplitudes and evaluated in the time and frequency domains to ensure integrity. During spatial mapping and calibration, signals were received by a MK301 microphone capsule (0.005–100 kHz, ± 2 dB; Microtech Gefell GmbH, Gefell, Germany) with a C617 body (Josephson Engineering, Santa Cruz, CA, USA) and BPS-1 power supply (Stewart Electronics, Rancho Cordova, CA, USA) and passed through the same filter and data acquisition hardware used for signal generation before being measured in HTP. Sound field mapping and daily calibration were conducted in the absence of the animal.

### Masked hearing thresholds

We measured masked hearing thresholds at the two frequencies with lowest absolute thresholds (800 Hz and 3200 Hz) in the presence of octave-band white noise centered at each test frequency. Critical ratios (CRs) were calculated as the difference between the SPL of the masked threshold and the power spectral density level of the masking noise (Fletcher 1940). Due to KE18’s elevated absolute thresholds, testing was limited to the two frequencies of best sensitivity where maximum signal level did not need to exceed 90 dB re 20 μPa during audiometry.^1^

The auditory masking paradigm was similar to that used for the audiogram, with the addition of masking noise paired with the onset of the trial light. Masking noise was generated and spectrally flattened with custom LabVIEW software (NI, Austin, TX, USA), projected through the computer sound card, and mixed with the test signal at a P1000 power amplifier (Hafler Professional, Tempe, AZ, USA) before reaching the JBL 2123H speaker. Masker duration was 8 s (500 ms rise/fall time) with the received spectral density level 10 dB above the corresponding absolute hearing threshold. Masked thresholds were measured using the same adaptive staircase procedure described earlier.

We spatially mapped the masking noise to ensure a stable sound field using the 14-position grid described previously. The 1/3-octave band levels comprising the octave-band masker were measured to confirm acceptable variability (≤ 6 dB) of all three bands across the mapping grid. Additionally, mapping confirmed that the center 1/3-octave band level measured at all 14 positions fell within 3 dB of that received at the daily calibration position. The masker was calibrated prior to each session to confirm that the center 1/3-octave band was within 1 dB of expected and that the surrounding 1/3-octave bands were within 3 dB of expected. During spatial mapping and calibration, masking noise was received through the Microtech microphone and analyzed with Spectra-PLUS software v.5.2.0.14 (Pioneer Hill Software LLC, Poulsbo, WA, USA) on a laptop computer.

### External ear morphology

To evaluate external ear anatomy in relation to what is known about hearing capabilities in the two Phocidae subfamilies, we conducted a simple comparison of the sizes of the external ear openings for the 18 extant true seal species. We used Adobe Illustrator v.23.1.1 (Adobe Inc., San Jose, CA, USA) to digitally trace a silhouette of each species from reference photos, including the meatal opening and other anatomical landmarks. Seals were hauled out or at the surface of the water in all photos.

## Results

### Audiogram

**Fig. 2:**
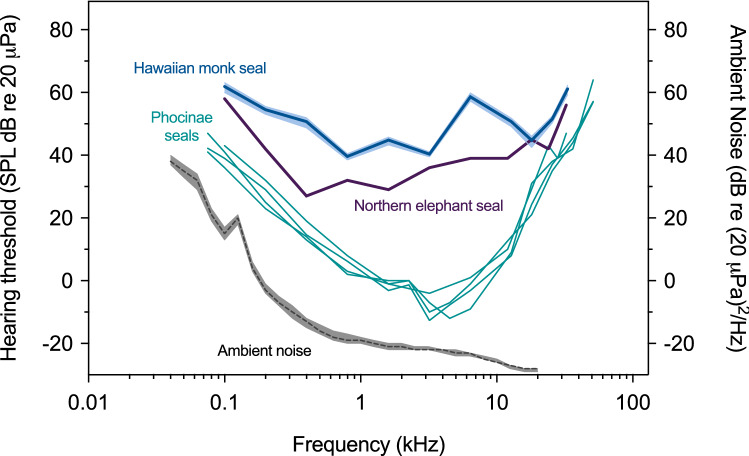
In-air audiogram for one Hawaiian monk seal, obtained using psychophysical methods; the shaded area around the audiogram depicts 95% confidence intervals. Associated hearing data are provided in Table 1. Ambient noise in the testing environment (dashed line corresponding to the right *y*-axis) is reported in terms of power spectral density levels; this noise curve is bounded by the 10th (above) and 90th (below) percentile statistics of the noise distribution. For comparison, audiograms are shown for representative species from each subfamily of true seals. Hearing data for the Phocinae subfamily include audiograms for harbor [*n* = 1, (Reichmuth et al. 2013)], spotted [*n* = 2, (Sills et al. 2014)], and ringed seals [*n* = 1, (Sills et al. 2015)]. For the Monachinae subfamily, data are only available for the northern elephant seal [*n* = 1, (Reichmuth et al. 2013)] (color online)

Absolute (unmasked) in-air hearing thresholds, false alarm rates, ambient noise levels, and threshold-to-noise offsets are provided for one Hawaiian monk seal in Table 1. The resulting audiogram and associated ambient noise floor, along with representative auditory data for Monachinae and Phocinae seals, are shown in Fig. 2. Psychometric functions for the thresholds are provided in Online Resource 2.

**Table 1:** In-air hearing thresholds and critical ratio measurements obtained for a Hawaiian monk seal using psychophysical methods

Frequency kHz	Absolute hearing thresholds					Critical ratios			
	Threshold dB re 20 µPa	95% confidence interval dB re 20 µPa	False alarm rate	Ambient noise dB re (20 µPa)^2^/Hz	Threshold-to-noise offset dB	Masked threshold dB re 20 µPa	Masker level dB re (20 µPa)^2^/Hz	Critical ratio dB	False alarm rate
0.1	62	60–63	0.22	15	47	–	–	–	–
0.2	55	53–56	0.12	− 3	57	–	–	–	–
0.4	51	49–52	0.08	− 13	64	–	–	–	–
0.8	40	38–41	0.11	− 19	58	72	50	23	0.16
1.6	45	43–46	0.05	− 21	65	–	–	–	–
3.2	40	39–41	0.22	− 22	62	78	50	28	0.18
6.4	59	57–60	0.25	− 23	82	–	–	–	–
12.8	51	49–52	0.17	− 27	78	–	–	–	–
18.1	45	42–46	0.23	− 28	73	–	–	–	–
25.6	52	50–52	0.11	–	–	–	–	–	–
33.2	61	59–62	0.17	–	–	–	–	–	–

Measured 50% detection thresholds are provided for 11 test frequencies, along with corresponding 95% confidence intervals, false alarm rates, ambient noise levels, and threshold-to-noise offsets. The psychometric functions associated with these thresholds are provided in Online Resource 2. Ambient noise levels are reported in units of power spectral density, calculated from the 1/3-octave band levels surrounding each test frequency. Threshold-to-noise offsets are given as the difference between hearing threshold and noise power spectral density. For the two frequencies where critical ratio measurements were made, masked 50% detection thresholds, masking noise spectral density levels, and false alarm rates are also provided

The audiogram lacked the characteristic U-shape of mammalian hearing curves and instead was relatively flat with a distinct elevation at 6.4 kHz. The functional range of hearing—the range of frequencies audible at 60 dB re 20 μPa (as in Heffner and Heffner 2007)—fell between 0.1 and 33 kHz, spanning almost eight octaves across the audiogram. The lowest threshold of 40 dB re 20 μPa was measured at 0.8 and 3.2 kHz, indicating relatively poor overall sensitivity to airborne sounds. The difference between the low- and high-frequency hearing limits and best hearing was only 20 dB. Additionally, the low- and high-frequency regions of the audiogram did not show pronounced declines in sensitivity; the low-frequency slope was approximately 7 dB per octave, while the high-frequency slope was approximately 18 dB per octave. Audiometric signals transitioned from inaudible (0% detection) to reliably detectable (100% detection) over a range of 6–10 dB.

The measured audiogram fell well above the ambient noise floor with threshold-to-noise offsets ranging from 47 to 82 dB. Repeated testing revealed differences of 3 dB or less for thresholds at both 6.4 and 12.8 kHz. KE18’s average false alarm rate throughout testing was 0.16 (range 0.05–0.25).

### Critical ratios

**Fig. 3:**
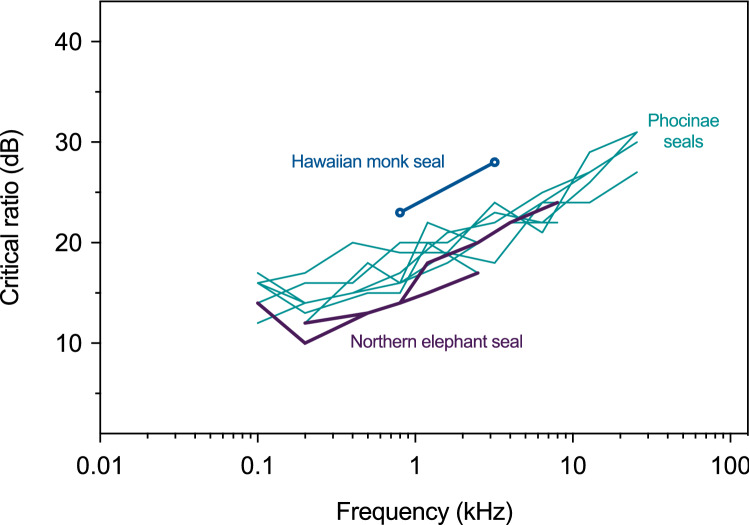
Critical ratio measurements for one Hawaiian monk seal at 0.8 and 3.2 kHz (open circles). For comparison, critical ratios are shown for representative species from each subfamily of true seals. Data are provided for bearded, *Erignathus barbatus* [*n* = 1, (Sills et al. 2020)], harbor [*n* = 1, (Southall et al. 2000, 2003)], ringed [*n* = 2, (Sills et al. 2015)], and spotted seals [*n* = 2, (Sills et al. 2014)] of the Phocinae subfamily. For the Monachinae subfamily, critical ratio measurements are only available for the northern elephant seal [*n* = 1, (Southall et al. 2000, 2003)] (color online)

Masked thresholds, masking noise levels, critical ratios, and false alarm rates evaluated at two frequencies are provided in Table 1. Critical ratios were 23 and 28 dB at 0.8 and 3.2 kHz, respectively. These data are shown in Fig. 3 with representative data from several Phocinae and Monachinae seal species. KE18’s average false alarm rate during these measurements was 0.17 (range 0.16–0.18).

### Comparative auditory anatomy

**Fig. 4:**
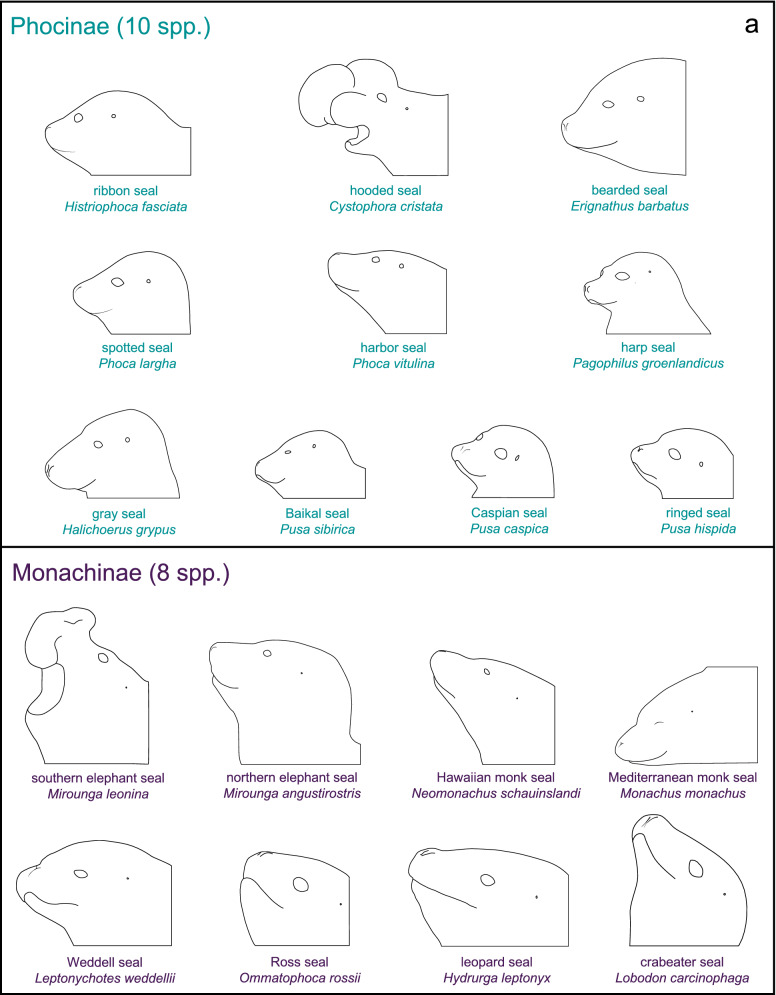

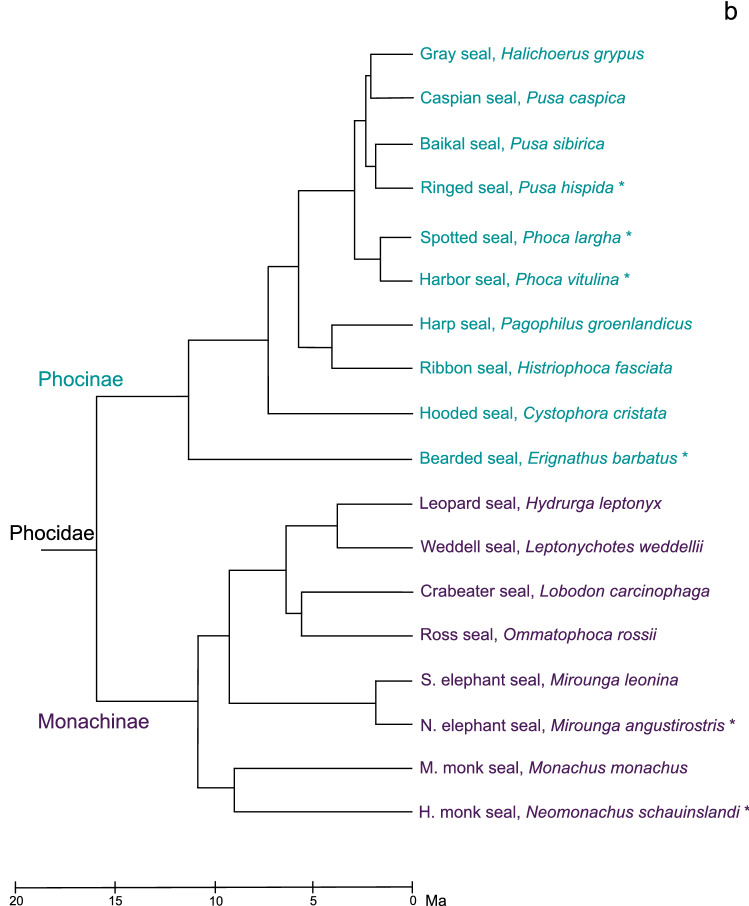
Silhouettes of the 18 extant seal species of family Phocidae and their phylogenetic relationships. **a** Evaluation of external auditory meatus opening size between the Monachinae and Phocinae subfamilies reveals that all Monachinae species have extremely small, likely occluded meatal orifices while those of the Phocinae species are comparatively large and open. Images were traced from reference photographs of seals while hauled out or at the water’s surface and are approximately scaled to reflect differences in head size across species. Illustrations are internally consistent, with accurate representation of the relative position and size of key facial features. **b** The scaled Phocidae phylogeny is adapted from Rule et al. (2020), with both subfamilies rotated at the first node so the earlier diverging species are at the base. Extinct lineages are not shown. Some details of the Phocidae phylogeny remain to be resolved (see Árnason et al. 2006; Higdon et al. 2007; Fulton and Strobeck 2010; Berta et al. 2018; Rule et al. 2020). The six seal species with recent behavioral hearing data (see Figs. 2 and 3) are marked with an asterisk (*)

Digitally illustrated silhouettes of all 18 extant true seals are provided in Fig. 4a for a comparison of the external auditory meatal opening size relative to other anatomical landmarks. This depiction shows that all Monachinae seals have an extremely small and essentially closed meatal orifice compared to the relatively larger meatal openings of Phocinae species. A phylogenetic representation of the Phocidae family, which references species for which recent audiometric data are available (Figs. 2 and Fig. 3), is provided in Fig. 4b.$\rightarrow$

## Discussion

This Hawaiian monk seal exhibited notably poor terrestrial hearing, with best sensitivity of 40 dB re 20 µPa and a range of functional hearing extending from 0.1 to 33 kHz. Hearing range was constrained in both the low- and high-frequency regions of the audiogram relative to that of Phocinae seals evaluated under the same conditions; best sensitivity was approximately 50 dB higher. The distinct upward notch at 6.4 kHz, which is also evident in the underwater audiogram of this individual (Sills et al. 2021), does not occur in other true seals. Overall, as observed for KE18’s underwater audiogram, this hearing curve does not correspond well with those of related species but best matches that of the northern elephant seal (Reichmuth et al. 2013)—the only other Monachinae seal for which auditory data are available.

The elevated thresholds of this individual compared to Phocinae seals cannot be explained by experimental conditions or animal behavior. High threshold-to-noise offsets indicate that ambient noise in the acoustic chamber did not influence measured hearing thresholds. Rather, auditory thresholds were well above noise levels at all frequencies. Typically, species-specific critical ratios are compared to threshold-to-noise offsets to determine whether thresholds could have been limited by background noise. As the only critical ratios available for Hawaiian monk seals are the two from this study, we used available data for other true seals for the remaining frequencies (Erbe et al. 2016; Sills et al. 2020). Threshold-to-noise offsets generously exceeded predicted critical ratios at each frequency (by 30–61 dB). In addition, KE18 did not exhibit an overly conservative response bias, which could have prevented the measurement of lower thresholds. Finally, the seal’s reliable behavior during testing and subsequent repeated testing at two frequencies confirms that his performance did not improve after additional experience with the task. Thus, the measurement of elevated absolute (unmasked) hearing thresholds cannot be attributed to insufficient practice on the behavioral task.

Critical ratios at 0.8 and 3.2 kHz were 3–10 dB higher than representative data from both true seal subfamilies. However, these values did increase with increasing frequency at a similar rate as for other true seals. Additional data are needed for a more complete comparison of masked hearing abilities, but further testing was not possible in this case. Our limited masking data suggest that Hawaiian monk seals may not have the same derived ability as other true seals to hear well in noisy environments.

These auditory data are available for only one subject, as is the case for the northern elephant seal (Reichmuth et al. 2013). Therefore, the high auditory thresholds measured for this seal across the frequency range of hearing could potentially be due to individual differences and not representative of all conspecifics. However, the poor terrestrial hearing of the two Monachinae seals tested thus far is consistent with the evolutionary biology of true seals, especially with respect to auditory anatomy.

### Relevance to auditory anatomy

Certain aspects of Hawaiian monk seal auditory anatomy may help explain the apparently reduced hearing sensitivity of this species. In particular, features of the external auditory meatus of Monachinae seals likely limit terrestrial auditory abilities; this opening can be qualitatively described as a pinhole that is often occluded with hair, making it essentially closed in air. Further, it is unknown whether the auditory canal is air filled or collapsed when these seals are resting on land. Thus, it seems reasonable to conclude that the ability of Monachinae seals to receive airborne sounds through the conventional terrestrial pathway is reduced (e.g., Kastak and Schusterman 1999). In contrast, the orifice of the external ear opening of Phocinae seals—who possess acute in-air hearing abilities—is large and surrounded by muscles that enable voluntary opening and closing of the channel leading to the auditory canal. When these seals are listening in air, this canal is thought to remain open and air filled, enabling hearing to occur efficiently through the conventional pathway (Møhl 1968). To put these anatomical differences into perspective, occluding the human meatal opening with finger, palm, or tragus causes a 25–45 dB reduction in hearing threshold (Holland 1967), enough to largely account for the elevated hearing thresholds observed in the two Monachinae seals evaluated thus far. A further reduction in terrestrial hearing ability may occur within the auditory canal, as King (1969) notes that the portion of the canal immediately behind the meatal opening has a longer unsupported section than in Phocinae species. Based on these anatomical considerations, it appears that the reception of airborne sounds may be constrained in all Monachinae seals by their peripheral auditory anatomy (as illustrated in Fig. 4a). These anatomical characteristics explain both elevated aerial hearing thresholds measured behaviorally in Hawaiian monk seals and northern elephant seals, as well as small-amplitude auditory-evoked potentials (AEPs) measured electrophysiologically in several Monachinae species, including northern and southern elephant seals, *Mirounga leonina* (Bornemann et al. 2007; Houser et al. 2007, 2008; Reichmuth et al. 2007) and leopard seals, *Hydrurga leptonyx* (Tripovich et al. 2011).

While many features of the middle ear are shared among true seals, there are anatomical indications that suggest a divergence between the two subfamilies. The ossicles of the middle ear are heavy and enlarged in northern elephant seals (Marsh 2001), a trait that could also influence sound conduction (if present) in other Monachinae seals. Another notable difference is the area ratio between the tympanic membrane and the oval window. Terrestrial hearing is improved when this ratio is large (i.e., the oval window is relatively small), because ossicular movement is amplified upon reaching the cochlea. More anatomical data are needed, but this ratio has been measured as 18–38:1 versus 9–10:1 in Phocinae and Monachinae species, respectively (Repenning 1972; King 1983). The higher tympanic membrane-to-oval window ratio in Phocinae seals is more similar to that of terrestrial Carnivores with sensitive in-air hearing (> 35:1; King 1983). While this area ratio cannot be used to estimate hearing sensitivity, a lower value for the Monachinae species suggests relatively poorer middle ear function with reduced hearing ability (Rosowski 1994; Mason 2016), as well as increased pressure tolerance during submersion (Repenning 1972). A final intriguing characteristic of the middle ear of Hawaiian monk seals concerns the distribution of cavernous tissue lining the middle ear cavity. Their particular distribution is notably similar to that of the distantly related otariid Carnivores (Repenning 1972; Repenning and Ray 1977) and can be considered a basal or more ‘primitive’ auditory trait (Wyss 1988).

In terms of skull morphology, the petrous bone—a pyramid-shaped portion of the temporal bone housing the inner ear—differs in monk seals relative to other true seals. Monk seals have a dorsoventrally flattened petrosal apex with a V-shaped outline in contrast to the hypertrophied bone at the petrosal apex of most other seals (Repenning and Ray 1977; Wyss 1988). In Hawaiian monk seals, the dorsal part of the petrosum is unexpanded; conversely, this surface is enlarged in Phocinae seals and may be linked to sensitive underwater hearing (Repenning and Ray 1977; Wyss 1988). This feature of the petrosum in Hawaiian monk seals is not only unique among extant seals, but among fossil species as well. Unlike other true seals, monk seals also have vestigial remnants of the petrosal lip roof of the internal auditory meatus (Wyss 1988), which suggests similarities to otarrid and odobenid Carnivores and may imply less derived auditory anatomy than other true seals. With respect to the cochlea, it has been noted that the basal whorl of the Hawaiian monk seal cochlea is relatively small compared to those of other seals (Repenning and Ray 1977); in addition, the distinct upward notch of this audiogram at 6.4 kHz suggests a cochlear anatomy that may be unique to the species. Together, these features of the inner ear and surrounding skull suggest that Hawaiian monk seals may have the least derived auditory anatomy of all true seals, which could help to explain the reduced sensitivity of this species to both airborne and waterborne sounds.

### Relevance to phylogenetic relationships

Observed anatomical differences between Phocidae subfamilies may not be unexpected given the evolutionary history of this group. Recently, fossils dating to the Pliocene (~ 3–5 mya) were discovered in New Zealand and identified as a new species of monk seal—the first monk seal ever found in the southern hemisphere (Rule et al. 2020). This finding contradicts the prevailing theory that monk seals evolved exclusively in the northern hemisphere. Instead, these new data suggest that all three Monachinae tribes coexisted in the southern hemisphere and that Monachinae evolution primarily occurred in isolation from the northern Phocinae seals. This discovery has profound impacts on our biogeographical understanding of true seal evolution. Analysis of the monk seal lineage based on both fossil and genetic evidence suggests that Hawaiian monk seals are the oldest species in this group (Rule et al. 2020), contrary to recent categorization of the Mediterranean monk seal as the earlier diverging species (Scheel et al. 2014). This new interpretation of monk seal evolution may explain the ‘primitive’ auditory anatomy observed in this species and, thus, their apparently reduced hearing abilities.

Because monk seals are the oldest branch within the Monachinae lineage, similarities in hearing ability with the more recently derived, deep-diving elephant seals suggest common selective pressures on ancestral species more than 12 million years ago. However, audiometric measurements obtained with additional Monachinae species are needed to confirm this idea. As most hearing data in marine mammals come from a few representative species, our unexpected findings for Hawaiian monk seals underscore the importance of sampling within and across phylogenetic clades to better understand auditory adaptations from an evolutionary perspective.

### Relevance to social communication

The auditory data reported in this study confirm that, while Hawaiian monk seals have poor sensitivity to airborne sounds in general, they are capable of detecting their own low-frequency vocalizations. However, their elevated thresholds indicate that terrestrial signaling probably occurs over relatively short ranges (as noted in Miller and Job 1992) or could rely on the production of high-amplitude calls. For example, male northern elephant seals overcome poor hearing sensitivity by emitting airborne calls that are among the loudest measured to date (Southall et al. 2019b). The source levels of Hawaiian monk seal airborne vocalizations have not yet been measured but do not seem to be of particularly high amplitude (Stacie Robinson, personal communication). It is possible that multimodal cues including acoustic, seismic, visual, or olfactory components (Miller and Job 1992) facilitate social communication during the extended breeding season in this species.

### Implications for conservation and management

The auditory measurements reported for this individual address significant gaps in our understanding of sensory systems—including the use of sound—in Hawaiian monk seals. From a management perspective, we note that the reported audiogram is captured by the Phocid Carnivores in Air (PCA) hearing group proposed in recent marine mammal noise exposure criteria (Southall et al. 2019a). Thus, the application of the PCA weighting function to predict potential noise effects is likely conservative for this species. However, elevated critical ratio measurements suggest that Hawaiian monk seals do not have efficient hearing in noise and therefore may be more susceptible to auditory masking. As critical ratios can be applied to predict masking in terrestrial and aquatic environments (see Richardson 1995; Reichmuth 2012; Erbe et al. 2016), these data enable estimation of auditory masking induced by natural and anthropogenic noise in both media.

This study advances knowledge of the acoustic ecology of Hawaiian monk seals, including auditory adaptations, evolutionary considerations, and social communication. While additional behavioral measurements describing auditory capabilities for this species and other Monachinae seals will be difficult and expensive to obtain, such data are needed to validate these findings and conclusions.

## Supplementary Information

Below is the link to the electronic supplementary material.

Supplementary file1 Examples of auditory go/no-go trials conducted with a Hawaiian monk seal in the acoustic chamber at Long Marine Laboratory, Santa Cruz, CA (M4V 212783 KB)

Supplementary file2 (PDF 277 KB)

## Data Availability

Not applicable.

## Footnotes

1 Levels required for testing at other frequencies were above those authorized by the National Marine Fisheries Service (marine mammal research permit 19590 to T. Williams).
