# Supplementary material for: In-air hearing in Hawaiian monk seals: implications for understanding the auditory biology of Monachinae seals
Source: J Comp Physiol A Neuroethol Sens Neural Behav Physiol. 2021 Jun 18;207(4):561–73. doi: 10.1007/s00359-021-01498-y (PMC8222047; doi:10.1007/s00359-021-01498-y)

Electronic Supplement: Online Resource 2

In-air hearing in Hawaiian monk seals: implications for understanding the auditory biology of Monachinae seals

Journal of Comparative Physiology A

Brandi Ruscher[1*], Jillian M. Sills, Beau P. Richter, Colleen Reichmuth

[1]Department of Ocean Sciences, University of California Santa Cruz, 115 McAllister Way, Santa Cruz, CA 95060, USA

*Corresponding author email: bruscher@ucsc.edu

Psychometric functions obtained for one Hawaiian monk seal at 11 frequencies in air, with percent correct detection on signal-present trials (y-axis) shown as a function of sound pressure level in dB re 20 µPa (x-axis). Probit analysis was used to fit these functions to the percent correct detection at each stimulus level presented (6-8 SPLs and 38-51 trials pooled from three final staircase sessions for each frequency). The dashed line represents the stimulus level corresponding to 50% correct detection, as measured using an inverse prediction

Psychometric functions for Hawaiian monk seal KE18 in air

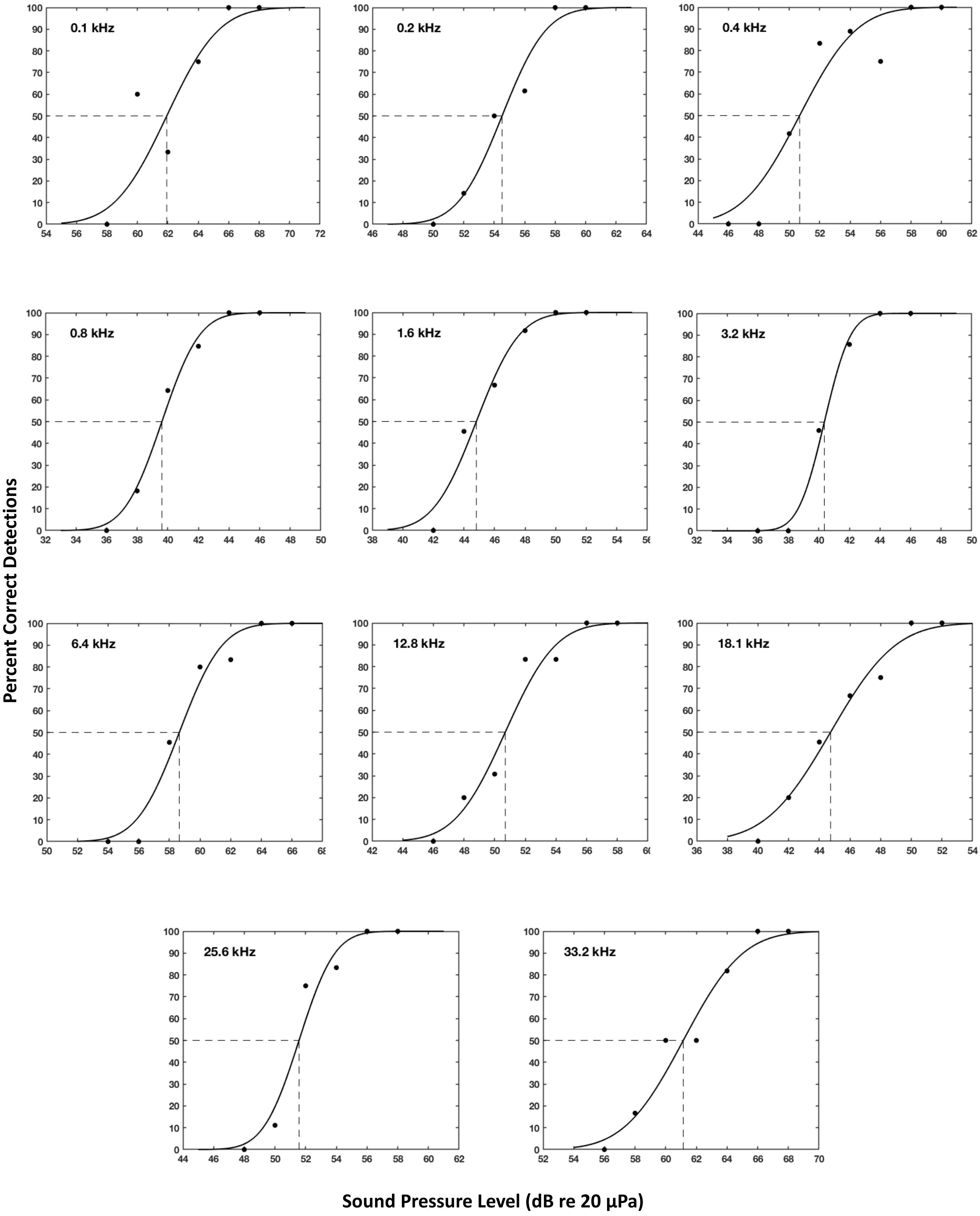

Supplement: Supplementary file 2 — Supplementary file2 (PDF 277 KB) [file 359_2021_1498_MOESM2_ESM.pdf]
